# A Study on Capacitive Micromachined Ultrasonic Transducer Periodic Sparse Array

**DOI:** 10.3390/mi12060684

**Published:** 2021-06-11

**Authors:** Tian Zhang, Wendong Zhang, Xingling Shao, Yuhua Yang, Zhihao Wang, Yang Wu, Yu Pei

**Affiliations:** 1 State Key Laboratory of Dynamic Testing Technology, North University of China, Taiyuan 030051, China; zhangtian0205@163.com (T.Z.); yangyuhua407@163.com (Y.Y.); wangxiaohaonuc@163.com (Z.W.); wuyang142222@163.com (Y.W.); 15513096651@163.com (Y.P.); 2 National Key Laboratory for Electronic Measurement Technology, School of Instrument and Electronics, North University of China, Taiyuan 030051, China

**Keywords:** CMUT, sparse array, ultrasound imaging, MEMS

## Abstract

Capacitive micromachined ultrasonic transducer (CMUT) is an ultrasonic transducer based on the microelectromechanical system (MEMS). CMUT elements are easily made into a high-density array, which will increase the hardware complexity. In order to reduce the number of active channels, this paper studies the grating lobes generated by CMUT periodic sparse array (PSA) pairs. Through the design of active element positions in the transmitting and receiving processes, the simulation results of effective aperture and beam patterns show that the common grating lobes (CGLs) generated by the transmit and receive array are eliminated. On the basis of point targets imaging, a CMUT linear array with 256 elements is used to carry out the PSA pairs experiment. Under the same sparse factor (SF), the optimal sparse array configuration can be selected to reduce the imaging artifacts. This conclusion is of great significance for the application of CMUT in three-dimensional ultrasound imaging.

## 1. Introduction

The CMUT ultrasonic array, based on MEMS technology, has attracted great attention from researchers, industry and clinical institutions. This is because the CMUT ultrasonic array is smaller and more sensitive than traditional ultrasonic components [1,2,3,4,5]. The research findings of Stanford are the most prominent when it comes to CMUT devices [6,7,8,9]. The North University of China has also paid great attention to the CMUT devices. We focused on the process structures used previously [10,11,12]. In recent years, the ultrasound imaging field has demanded greater imaging clarity, especially for the three-dimensional ultrasound imaging [13]. The requirements for the CMUT array elements, such as spacing, elements size, and element number, are also higher. The number of array elements can reach the hundreds or even thousands, resulting in high hardware complexity [14]. To solve this problem, researchers have provided a variety of strategies to design the sparse array [15,16,17,18,19]. This has included reducing the side-lobes caused by sparse array through optimization theories [20,21]. In reference [20], a new cost function was introduced to optimize the weighting coefficients of the elements, and a new annealing-based algorithm was proposed to compute the lowest cost solutions. However, the position of sparse array elements designed by these optimization theories is irregular, resulting in an uneven scanning line, which may affect the imaging quality. In this paper, the PSA pairs are designed based on the Vernier arrays [15]. Vernier arrays take advantage of the periodicity of the array elements: set p is a positive integer and d is array elements spacing; if the transmit aperture array elements spacing is (p−1)d and the receive aperture array elements spacing is pd, the overall effective aperture with the array elements spacing of d can be generated by convolution operation [22]. To make the effective aperture of Vernier arrays closer to the ideal effective aperture, in [23,24], a weighting method is proposed to ensure the signal-to-noise ratio (SNR) and reduce the grating lobes. In references [20,22,25,26], the studies of sparse array were limited to algorithm derivation and simulation imaging and had not been verified by experimental imaging. CMUT is the new generation of ultrasonic component [7], and its emission principle is different from that of a traditional piezoelectric transducer. This paper studies the analysis method of [25] to study the optimal PSA pairs in CMUT array imaging. It sets the array elements spacing to an arbitrary value (not limited to the (p−1)∗p), but the periodicity is guaranteed. For the CMUT arrays with the same SF, the optimal sparse array configuration is selected by comparing the effective aperture and beam patterns; the point targets imaging and CMUT linear array imaging are verified and the CMUT array experimental verification is carried out according to the criteria derived in [25]. Furthermore, Kaiser window apodization is performed on the optimal sparse array during the process of CMUT ultrasonic transmission, which further reduce the artifacts of CMUT sparse array imaging.

This article is organized as following. In Section 2, the structure of CMUT is introduced. In Section 3, CMUT sparse model and simulation are explained. Section 4 presents the selection of optimal sparse array configuration. Section 5 shows imaging verification. Section 6 concludes this article.

## 2. Structure of CMUT

**Figure 1:**
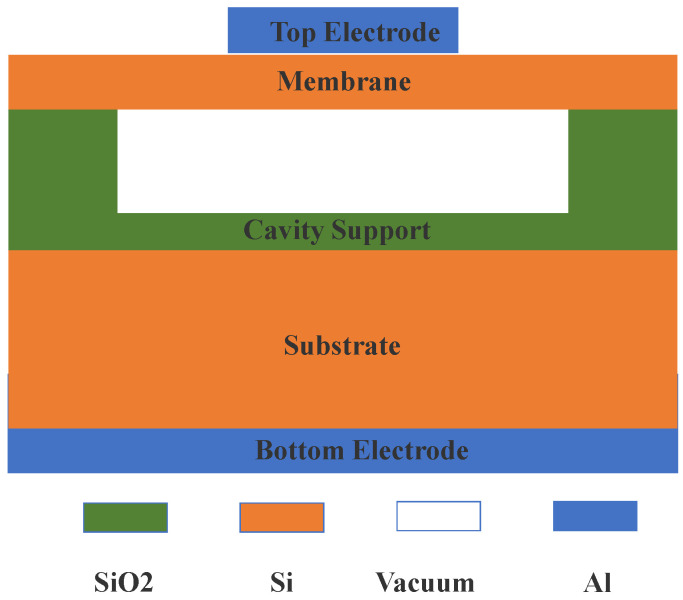
Structure of CMUT cell.

The CMUT component is based on a centralized diaphragm structure and consists of many parallel cells. The cell structure from top to bottom is: top electrode, membrane; cavity support, substrate, bottom electrode, and a vacuum cavity in the middle, as shown in Figure 1. The top electrode and bottom electrode, made of aluminum material, are used to connect the external electric signal and grounding. There is a superposition effect between cells. At a given resonance frequency, cells can vibrate simultaneously to produce ultrasonic waves, or they can vibrate under the action of ultrasonic waves, and then through a specific circuit to produce voltage signals. When a CMUT is in a working state, direct current (DC) bias voltage is applied to the two electrodes of the cells. For example, when the CMUT is in the receiving state, the DC is applied to the top electrode, and the electrostatic force causes the membrane to stretch downward until the electrostatic force and membrane resilience reaches a dynamic balance [27]. Under the action of external ultrasonic signal, the membrane vibrates and changes the capacitance between the top electrode and bottom electrode. This makes the output charge change and generates a weak induced current under the action of DC bias. The CMUT-induced current formula is [28]

(1) $$i_{CMUT}=V_{DC}\frac{C{\left(t\right)}^{2}}{\varepsilon_{0}A}\frac{\partial d(t)}{\partial t}$$

where $V_{DC}$ is the DC bias voltage, the unit is V. C(t) is the capacitance of the CMUT, the unit is nF. $\varepsilon_{0}$ is the dielectric constant of vacuum. A is the area of vibration membrane, the unit is mm^2^. $\frac{\partial d(t)}{\partial t}$ is the speed of vibration membrane. It can be seen from Formula (1) that $V_{DC}$ is proportional to $i_{CMUT}$, so in the appropriate range, DC should be increased to improve the output current of CMUT.

**Figure 2:**
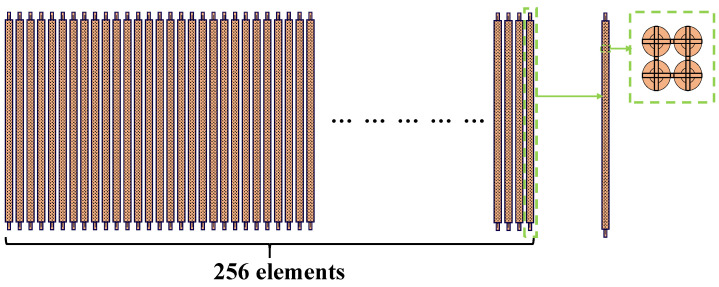
Structure of CMUT linear array.

The structure diagram of CMUT linear array is shown in Figure 2, and the array parameters are shown in Table 1.

## 3. CMUT Sparse Model and Simulation

### 3.1. CMUT Sparse Model

**Figure 3:**
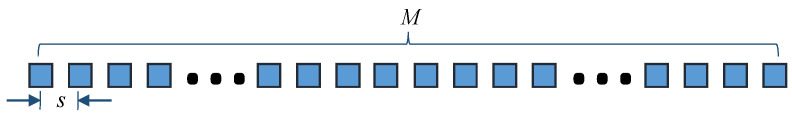
Conventional phased array of CMUT.

Figure 3 shows the conventional phased array (CPA) of CMUT, which uses all elements to transmit and receive. The one-way beam pattern can be expressed as [28]

(2) $$D_{CPA}(\theta )=\frac{\sin[(Mks\sin\theta )/2]}{\sin[(ks\sin\theta )/2]}$$

where M is the number of CPA array elements, k is the wave number, s is the center distance of array elements, and θ is the deflection angle.

The condition for the existence of grating lobes is

(3) $$\left|D_{CPA}(ks\sin\theta )\right|=\left|D_{CPA}(ks\sin\theta \pm 2n\pi )\right|$$

when kssinθ=0, i.e., θ=±2nπ(n=1,2,⋅⋅⋅), the position of grating lobes can be obtained as follows:

(4) $$\theta_{n}=\arcsin(\pm \frac{n\lambda }{s}),\begin{array}{ccc} & & n=1,2,\cdot \cdot \cdot \end{array}$$

**Figure 4:**
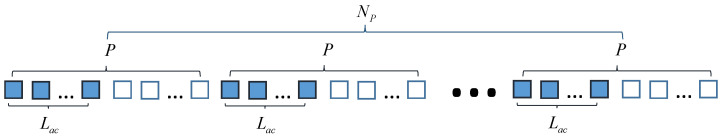
Periodic sparse model of CMUT linear array.

Figure 4 is the periodic sparse model of CMUT linear array. The total number of array elements is $N_{P}\times P$, the array elements are divided into $N_{P}$ periods, and each period has P elements. The number of active elements is $L_{ac}$. The array SF is defined as $SF=L_{ac}/P$. It should be noted that the active elements are continuous. According to Formula (4), when the grating lobes position of the transmitting array and the receiving array are the same, the grating lobes of the two-way beam pattern at the same position will occur, which is called the common grating lobes (CGLs). The purpose of this article is to eliminate CGLs by optimizing the layout of PSA pairs. Through the design of transmit sparse array (TSA) and receive sparse array (RSA), CGLs can be eliminated completely.

There are three criteria of CGLs elimination [25]:

(1) $\mathrm{TSA}\;(P_{T},1)/\mathrm{RSA}\;(P_{R},1)$, when the greatest common divisor (GCD) of $P_{T}$ and $P_{R}$ is 1;

(2) $\mathrm{TSA}\;(P_{T},L_{T}=P_{R})/\mathrm{RSA}\;(P_{R},1)$, when the GCD of $P_{T}$ and $P_{R}$ is $P_{R}$, where $P_{R}\leq P_{T}$;

(3) $\mathrm{TSA}\;(P_{T},l_{0})/\mathrm{RSA}\;(P_{R},1)$ or $\mathrm{TSA}\;(P_{T},1)/\mathrm{RSA}\;(P_{R},l_{0})$, where $l_{0}<\min(P_{T},P_{R})$. 

TSA/RSA pairs satisfying any one of the three criteria can eliminate CGLs in the two-way beam patterns.

### 3.2. Effective Aperture Comparison

**Figure 5:**
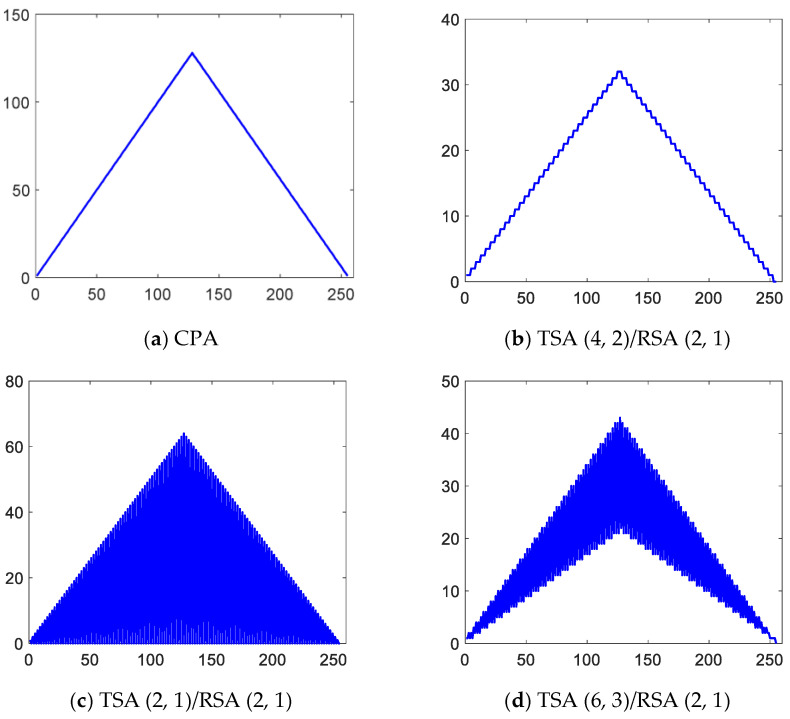
Comparison of effective aperture.

Take the SF of transmit array $SF_{T}=1/2$, and the SF of receive array $SF_{R}=1/2$, namely $SF_{two-way}=SF_{T}\times SF_{R}=1/4$. The CMUT sparse array configurations are: TSA (4, 2)/RSA (2, 1), TSA (2, 1)/RSA (2, 1), TSA (6, 3)/RSA (2, 1). TSA (2, 1)/RSA (2, 1) does not satisfy the criteria of CGLs elimination, yet TSA (4, 2)/RSA (2, 1) and TSA (6, 3)/RSA (2, 1) do satisfy the criteria of CGLs elimination. Figure 5a shows the effective aperture of CPA, which is a standard triangle [31]. Figure 5b–d shows the effective aperture of TSA (4, 2)/RSA (2, 1), TSA (2, 1)/RSA (2, 1), and TSA (6, 3)/RSA (2, 1), respectively. It can be seen that the effective aperture of TSA (2, 1)/RSA (2, 1) has the largest oscillating pattern, and the oscillating pattern range of TSA (6, 3)/RSA (2, 1) is reduced. The oscillating range of TSA (4, 2)/RSA (2, 1) is the smallest, which is the closest to the effective aperture of CPA. It can be preliminarily judged that TSA (4, 2)/RSA (2, 1) should be selected when the SF of the transmit array and receive array are both 1/2. 

### 3.3. Beam Patterns Comparison

**Figure 6:**
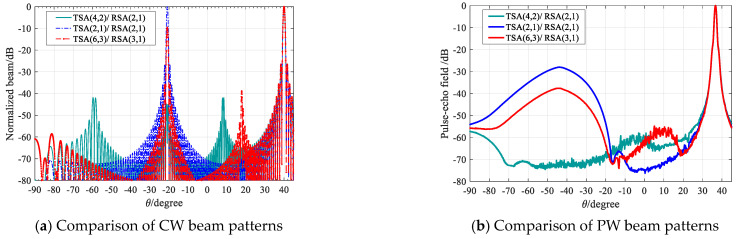
Comparison of beam patterns.

In this section, the continuous wave (CW) beam patterns and pulse wave (PW) beam patterns comparisons are performed on TSA (2, 1)/RSA (2, 1), TSA (4, 2)/RSA (2, 1), and TSA (6, 3)/RSA (2, 1). To make the side-lobes comparison clearer, the beam patterns deflection is 40°. Figure 6a shows the CW beam patterns comparison diagram. It can be seen that at the position of −20°, TSA (2, 1)/RSA (2, 1) has the highest grating lobe, whereas the grating lobe of TSA (6, 3)/RSA (2, 1)) is reduced, though it still reaches the height of −10 dB. TSA (4, 2)/RSA (2, 1), however, reduces the grating lobe to less than −40 dB in the same position. Figure 6b shows the PW beam patterns comparison diagram simulated in Field II. The side-lobe levels of the PW beam patterns are lower than those of the CW beam patterns by no less than 30 dB; it can be seen that the side-lobes appear at −20°, the side-lobe of TSA (2, 1)/RSA (2, 1) is still the highest, while the side-lobe of TSA (6, 3)/RSA (2, 1) decreased by 10 dB. However, the side-lobe of TSA (4, 2)/RSA (2, 1) is the lowest. The comparison of PW beam pattern results is consistent with that of CW beam patterns.

This section studies the sparse array configurations which satisfy the criteria of CGLs elimination can reduce the side-lobe of beam patterns under the same SF. In the following section, we will study the selection of the optimal sparse array configuration through the beam patterns and perform imaging verification.

## 4. Selection of Optimal Sparse Array Configuration

This section takes $SF_{two-way}=1/6$ as an example. There are five sparse array configurations that satisfy the criteria of CGLs elimination: TSA (3, 1)/RSA (2, 1), TSA (6, 2)/RSA (2, 1), TSA (6, 3)/RSA (3, 1), TSA (6, 4)/RSA (4, 1), TSA (6, 5)/RSA (5, 1). 

**Figure 7:**
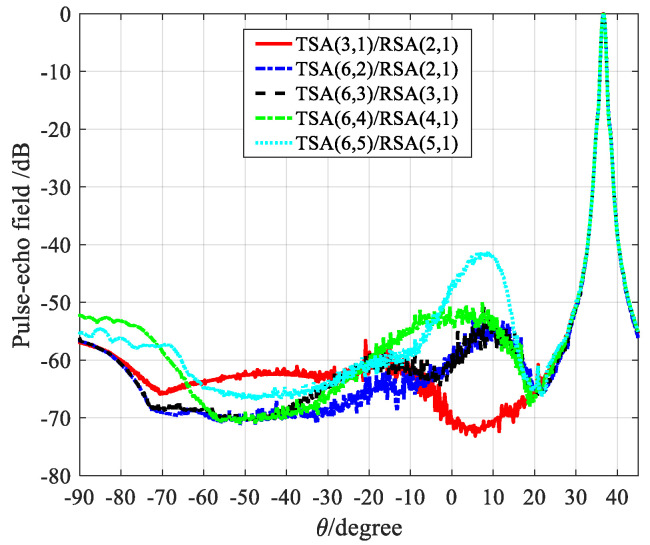
PW beam patterns comparison for $SF_{two-way}=1/6$.

Figure 7 shows the comparison result of the PW beam patterns. It can be seen that the side-lobe levels of TSA (6, 4)/RSA (4, 1) and TSA (6,5)/RSA (5,1) are higher, while TSA (3, 1)/RSA (2, 1), TSA (6, 2)/RSA (2, 1), TSA (6, 3)/RSA (3, 1) have relatively lower side-lobe levels.

For a quantitative comparison of the five sparse array configurations in Figure 7, Table 2 provides the mean side-lobe level and the peak side-lobe level in the region −30° < θ < 30° of each sparse array configuration in Figure 7. Table 2 shows that the side-lobe levels of TSA (6, 4)/RSA (4, 1) and TSA (6, 5)/RSA (5, 1) are higher, and although there is no significant difference in the side-lobe levels of TSA (3, 1)/RSA (2, 1), TSA (6, 2)/RSA (2, 1), and TSA (6, 3)/RSA (3, 1), it can be seen that the TSA (3, 1)/RSA (2, 1) has the lowest the peak side-lobe level and mean side-lobe level. Therefore, TSA (3, 1)/RSA (2, 1) should be selected as the optimal sparse array configuration.

## 5. Verification

### 5.1. Simulation Verification

**Figure 8:**
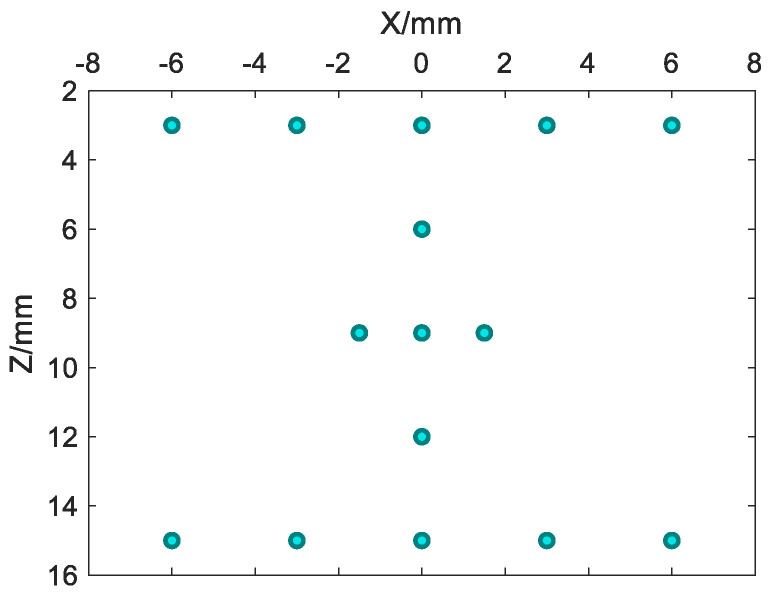
Point targets space position.

To verify the imaging effect of the optimal PSA pairs, point targets imaging are carried out. In point targets imaging, 15 spatial points are set on the Oxz plane, as shown in Figure 8. The coordinates are: (−6.00 mm, 0.00 mm, 3.00 mm), (−3.00 mm, 0.00 mm, 3.00 mm), (0.00 mm, 0.00 mm, 3.00 mm), (3.00 mm, 0.00 mm, 3.00 mm), (6.00 mm, 0.00 mm, 3.00 mm), (0.00 mm, 0.00 mm, 6.00 mm), (−1.50 mm, 0.00 mm, 9.00 mm), (0.00 mm, 0.00 mm, 9.00 mm), (1.50 mm, 0.00 mm, 9.00 mm), (0.00 mm, 0.00 mm, 12.00 mm), (−6.00 mm, 0.00 mm, 15.00 mm), (−3.00 mm, 0.00 mm, 15.00 mm), (0.00 mm, 0.00 mm, 15.00 mm), (3.00 mm, 0.00 mm, 15.00 mm), (6.00 mm, 0.00 mm, 15.00 mm).

**Figure 9:**
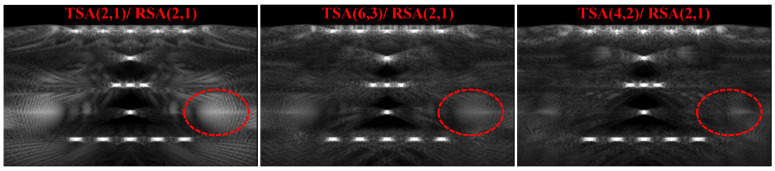
Point targets imaging comparison of $SF_{two-way}=1/4$.

Figure 9 shows the point targets imaging comparison results of $SF_{two-way}=1/4$, in which the TSA (2, 1)/RSA (2, 1) does not satisfy the criteria of CGLs elimination. As shown by the red dotted line, TSA (2, 1)/RSA (2, 1) in Figure 9 shows that the point targets’ imaging artifacts brightness is the highest, while TSA (6, 3)/RSA (2, 1) and TSA (4, 2)/RSA (2, 1) with the same SF can effectively reduce the brightness of artifacts because they satisfy the criteria of CGLs elimination. Compared with TSA (4, 2)/RSA (2, 1) and TSA (6, 3)/RSA (2, 1), TSA (4, 2)/RSA (2, 1) has fewer artifacts, which preliminarily verifies the validity of beam patterns. In order to quantitatively compare the point targets imaging results of $SF_{two-way}=1/4$, and taking the point targets imaging result of CPA as the standard, the mean square error (MSE) and peak signal-to-noise ratio (PSNR) [32] are compared. The comparison results are shown in Table 3. It can be seen from the quantitative comparison results that, under the same $SF_{two-way}=1/4$, the imaging result of TSA (4, 2)/RSA (2, 1) is the best.

**Figure 10:**
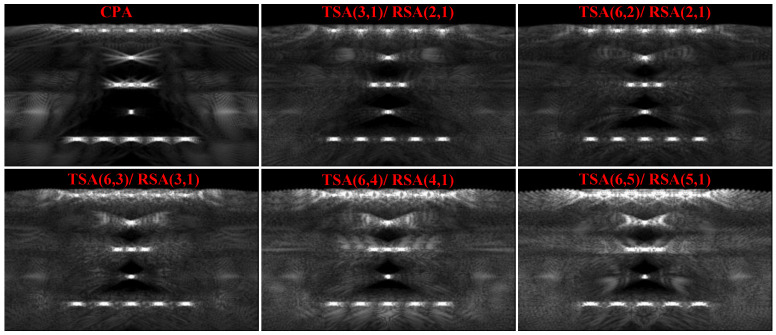
Point targets imaging comparison of $SF_{two-way}=1/6$.

Figure 10 shows the point targets imaging comparison results of $SF_{two-way}=1/6$. All sparse array configurations satisfy the criteria of CGLs elimination. It can be seen that TSA (6, 4)/RSA (4, 1) and TSA (6, 5)/RSA (5, 1) have a higher artifacts brightness. Compared with the three sparse array configurations TSA (3, 1)/RSA (2, 1), TSA (6, 2)/RSA (2, 1) and TSA (6, 3)/RSA (3, 1), TSA (3, 1)/RSA (2, 1) has the lowest artifacts brightness relative to TSA (6, 2)/RSA (2, 1) and TSA (6, 3)/RSA (3, 1), which can be used as the optimal sparse array configuration. In the same way, quantitative comparison results of point targets imaging under the same $SF_{two-way}=1/6$ are performed in Table 4. This conclusion verifies the beam patterns of Figure 7.

The simulation results of point targets imaging show that under the same SF, the sparse array configurations that satisfy the criteria of CGLs elimination can reduce side-lobes and artifacts, and thereby reduce hardware complexity. It is preliminarily concluded that the beam patterns can be used to select the optimal sparse array configuration.

### 5.2. Experimental Verification

**Figure 11:**
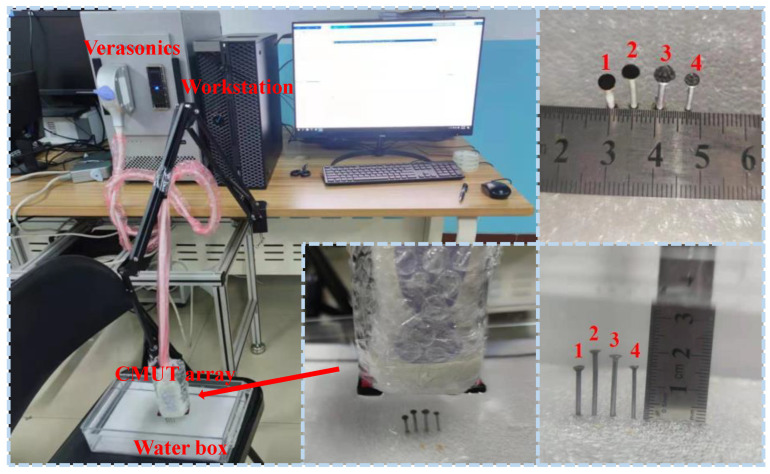
Experimental platform.

In this paper, a 256-element CMUT linear array is used for experimental verification. The parameters of CMUT linear array are shown in Table 1; the alternating voltage (AC) of CMUT linear array is 13 V in this experiment. The experimental platform is shown in Figure 11. The experiment is carried out on the Verasonics ultrasound platform [33,34,35]; four nails are fixed on the foam pad, using the CMUT linear array to image these four nails underwater. Each nail’s number is shown in the figure; the heights of nail 1, nail 2, nail 3, nail 4 are 15.00 mm, 20.00 mm, 19.00 mm, 16.00 mm, respectively. The distance between nail 1 and nail 2 is 5.00 mm, the distance between nail 2 and nail 3 is 6.00 mm, and the distance between nail 3 and nail 4 is 6.00 mm. In this section, two groups of experiments $SF_{two-way}=1/4$ and $SF_{two-way}=1/6$ are carried out, and the sparse array configurations are the same as that of point targets imaging.

#### 5.2.1. Criteria of CGLs Elimination Experimental Verification ($SF_{two-way}=1/4$)

In order to verify that the sparse array configuration under the same SF satisfies the criteria of CGLs elimination can reduce beam side-lobes and artifacts, experiments are carried out on the sparse array configuration of $SF_{two-way}=1/4$.

**Figure 12:**
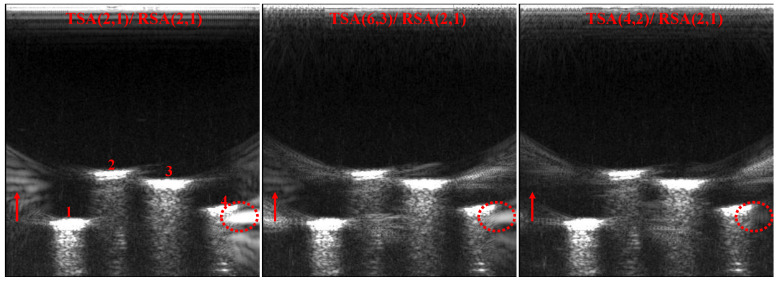
Imaging results of different CMUT sparse array configurations ($SF_{two-way}=1/4$).

Figure 12 shows the imaging comparison of TSA (2, 1)/RSA (2, 1), TSA (6, 3)/RSA (2, 1) and TSA (4, 2)/RSA (2, 1). From the TSA (2, 1)/RSA (2, 1) imaging result, the red circle and arrow indicate that the artifacts are obvious, especially the artifacts at the red circle, which cause the border of the nail 4 to be blurred. The artifacts brightness of TSA (6, 3)/RSA (2, 1) is lower than TSA (2, 1)/RSA (2, 1). The artifacts of TSA (4, 2)/RSA (2, 1) is almost disappeared, and the outline of nail cap can be clearly seen.

#### 5.2.2. Experimental Verification of Optimal Sparse Array Configuration Selection ($SF_{two-way}=1/6$)

**Figure 13:**
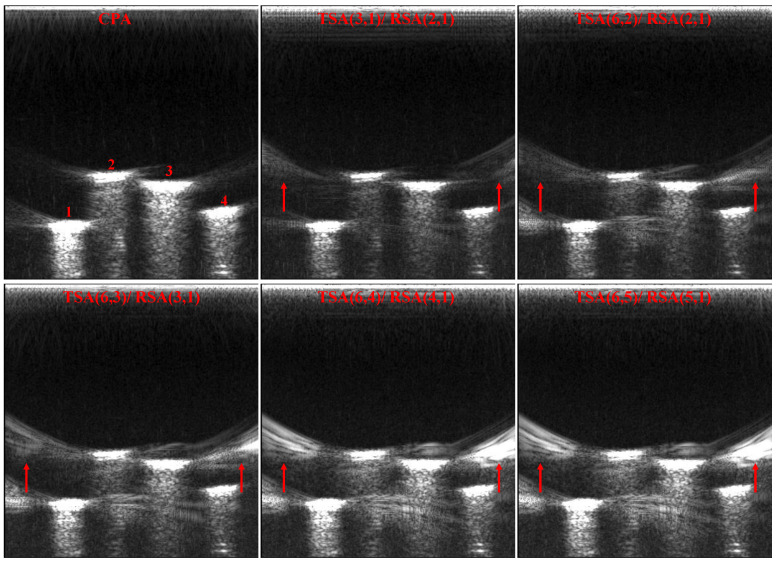
Imaging results of different CMUT sparse array configurations ($SF_{two-way}=1/6$).

The CMUT linear array is used to perform imaging experiments on all sparse array configurations (satisfy the criteria of CGLs elimination) of $SF_{two-way}=1/6$, the imaging comparison results are shown in Figure 13. Consistent with the beam patterns and simulation imaging results, the artifacts of TSA (3, 1)/RSA (2, 1) are the least in these sparse array configurations, and the artifacts of TSA (6, 5)/RSA (5, 1) have the highest artifacts brightness, almost the same as the brightness of nails. Imaging comparison results show that, for a CMUT array with the same SF, different sparse array configurations have great differences in imaging quality. Therefore, it is very effective to use the beam patterns to select the optimal sparse array configuration before actual imaging. 

**Figure 14:**
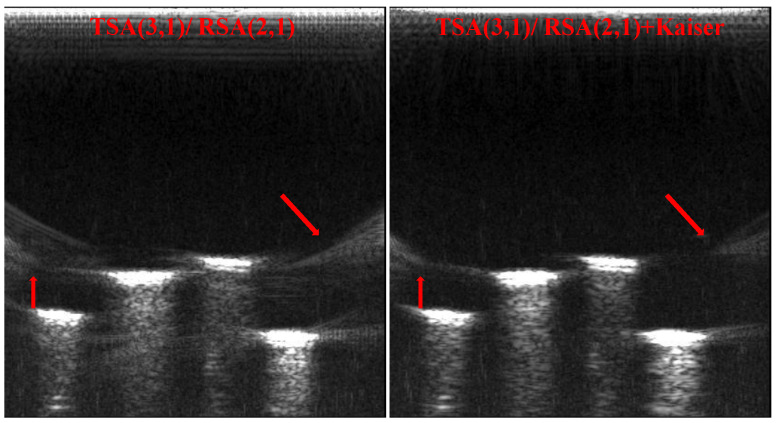
Imaging results comparison of Kaiser window apodization.

From the imaging comparison results, it can be seen that selecting the optimal sparse array configuration can reduce artifacts. To further improve the imaging effect of sparse array and produce similar results to the imaging effect of CPA, the Kaiser window apodization [36,37,38] is added during the ultrasonic transmitting process of CMUT array. Selecting the optimal array configuration TSA (3, 1)/RSA (2, 1) and Kaiser window [39,40,41] parameter β=8, Figure 14 shows the comparison diagram of imaging results. It is obvious that the artifacts at the point indicated by the arrow are significantly reduced, and the imaging contrast of the nails is increased. This experiment shows that by further improving the imaging process of optimal sparse array configuration, the imaging effect of the CMUT sparse array is closer to that of CPA on the basis of reducing hardware complexity.

## 6. Conclusions

In this paper, the PSA of CMUT linear array is studied. The effective aperture and beam patterns are used to preliminarily verify the criteria of CGLs elimination. Under the condition of the same SF, the optimal sparse array configuration is selected and verified by point targets imaging and experimental imaging. The imaging results show that the periodic sparse method in this article can not only reduce the hardware complexity, but also render the imaging quality very close to that of CPA. In particular, the experimental results of CMUT linear array prove the feasibility of PSA pairs, which is of great significance for the application of CMUT in the field of three-dimensional ultrasound imaging.

## Figures and Tables

**Table 1:** Parameters of CMUT linear array (KOLO [29,30], L22-8v).

Parameter Term	Value
Channels of elements	256
Center frequency	15.00 MHz
Bandwidth	(8.00 MHz, 22.00 MHz)
Element kerf	0.0377 mm
Element size	0.0703 mm × 2.50 mm
DC bias voltage	90.00 V

**Table 2:** Comparisons of mean and peak side-lobe levels in the region −30° < θ < 30° for different sparse array configurations.

Sparse Array Configuration	Mean (dB)	Peak (dB)
TSA (3, 1)/RSA (2, 1)	−58.98	−59.03
TSA (6, 2)/RSA (2, 1)	−58.43	−54.39
TSA (6, 3)/RSA (3, 1)	−57.24	−53.84
TSA (6, 4)/RSA (4, 1)	−55.64	−50.73
TSA (6, 5)/RSA (5, 1)	−53.70	−41.35

**Table 3:** Point-targets imaging quantitative comparison of $SF_{two-way}=1/4$.

Point-Targets Imaging	MSE	PSNR
TSA (2, 1)/RSA (2, 1)	4369.84	24.33
TSA (6, 3)/RSA (2, 1)	3656.92	30.40
TSA (4, 2)/RSA (2, 1)	1755.90	33.89

**Table 4:** Point targets imaging quantitative comparison of $SF_{two-way}=1/6$.

Point-Targets Imaging	MSE	PSNR
TSA (3, 1)/RSA (2, 1)	3795.83	34.22
TSA (6, 2)/RSA (2, 1)	3837.70	34.07
TSA (6, 3)/RSA (3, 1)	3975.23	33.92
TSA (6, 4)/RSA (4, 1)	4115.12	33.77
TSA (6, 5)/RSA (5, 1)	4257.40	27.74
